# A Rare Case of a Primary Cutaneous Collision Tumor Comprising Malignant Melanoma and Rhabdomyosarcoma

**DOI:** 10.7759/cureus.58910

**Published:** 2024-04-24

**Authors:** Cameron Gerhold, Connor Stonesifer, Dong-Lin Xie, Robert Norman

**Affiliations:** 1 Department of Medicine, Florida State University College of Medicine, Tallahassee, USA; 2 Department of Dermatology, University of Miami, Miami, USA; 3 Department of Dermatopathology, Tampa Community Hospital, Tampa, USA; 4 Department of Dermatology, Nova Southeastern University, Fort Lauderdale, USA

**Keywords:** dermatology, dermatopathology, rhabdomyosarcoma, melanoma, collision tumor

## Abstract

This case reports a 35-year-old man who presented with a painful erythematous nodule on his right posterior calf. He first noticed this nodule several years ago and it often bled upon contact with clothing. An excisional biopsy of the skin lesion revealed two distinct populations of cells. One population of epithelioid cells stained positive for Mart-1, HMB45, and SOX-10, confirming the diagnosis of malignant melanoma. The second population of cells stained positive for desmin and calponin, confirming the diagnosis of sarcoma with muscular differentiation. Subsequently, these unusual findings led to the diagnosis of a collision tumor comprising malignant melanoma and rhabdomyosarcoma. Follow-up PET/CT and brain MRI revealed no metastasis from the primary skin lesion. This case highlights a rare combination of cell types found within a collision tumor in addition to providing details on how to diagnose this skin lesion.

## Introduction

Collision tumors are neoplastic lesions composed of two histopathologically distinct tumor types. While collision tumors are an uncommon entity in dermatology, the most frequently observed cutaneous collision tumors involve basal cell carcinoma and melanocytic nevi [1,2]. We present a rare case of a collision tumor composed of malignant melanoma and rhabdomyosarcoma.

## Case presentation

A 35-year-old man with no past medical history presented to the clinic for evaluation of an erythematous nodule on the right posterior calf. He first noticed his lesion two years ago and felt it looked like a mole at that time. A few months prior to presentation, he reported that the lesion had grown into a large, inflamed nodule. The lesion was severely painful and frequently caught on clothing, causing it to bleed. There was no clinical history of other lesions, limb edema, or lymphadenopathy. He denied notable exposures and denied other symptoms, including fever, chills, fatigue, weight loss, and night sweats. He had no prior history of skin cancer. He reported a significant history of sun exposure to the affected area and rarely applied sunscreen.

**Figure 1:**
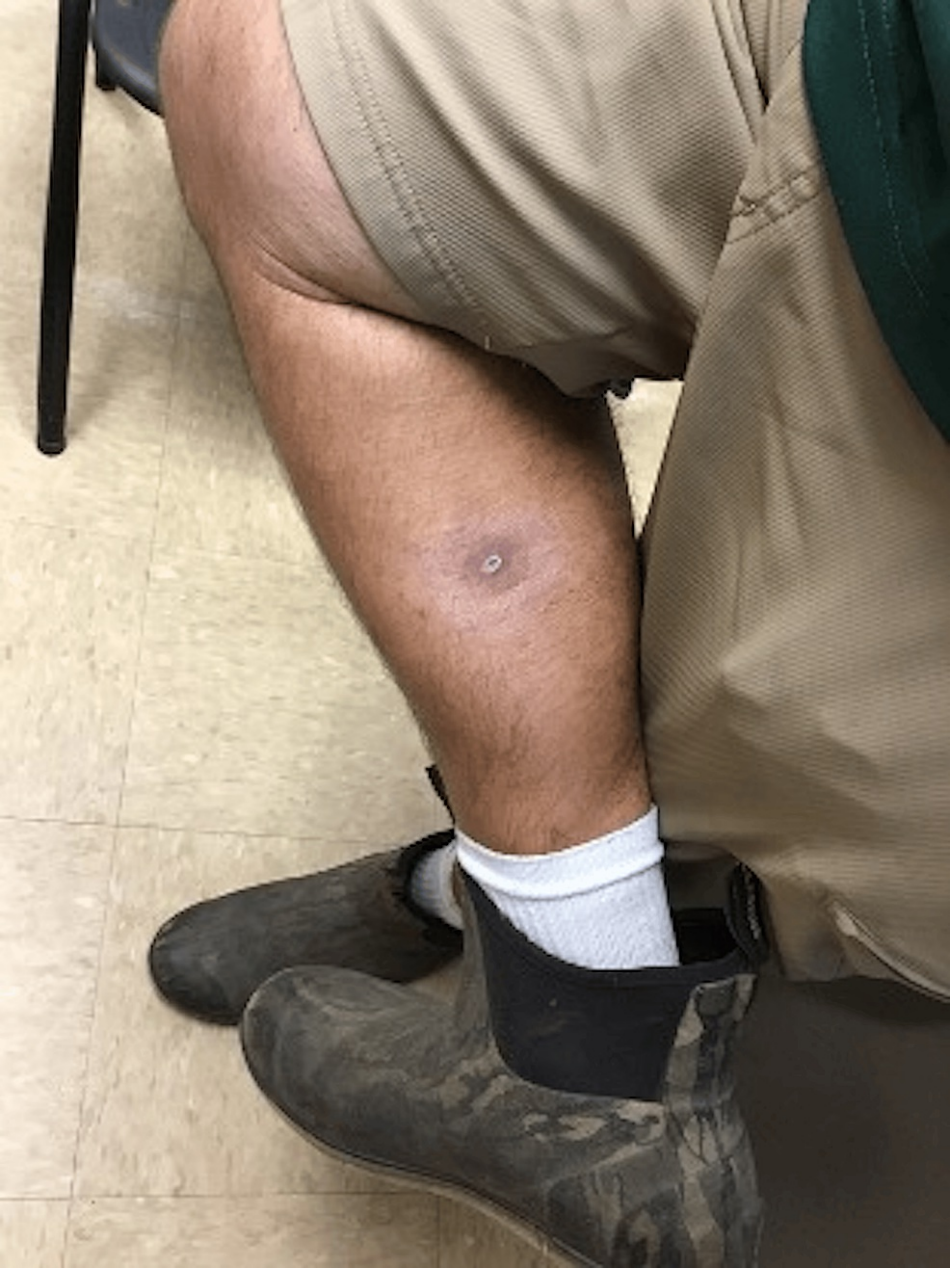
Clinical photograph of the ulcerated nodule with surrounding erythema on the right posteromedial calf.

**Figure 2:**
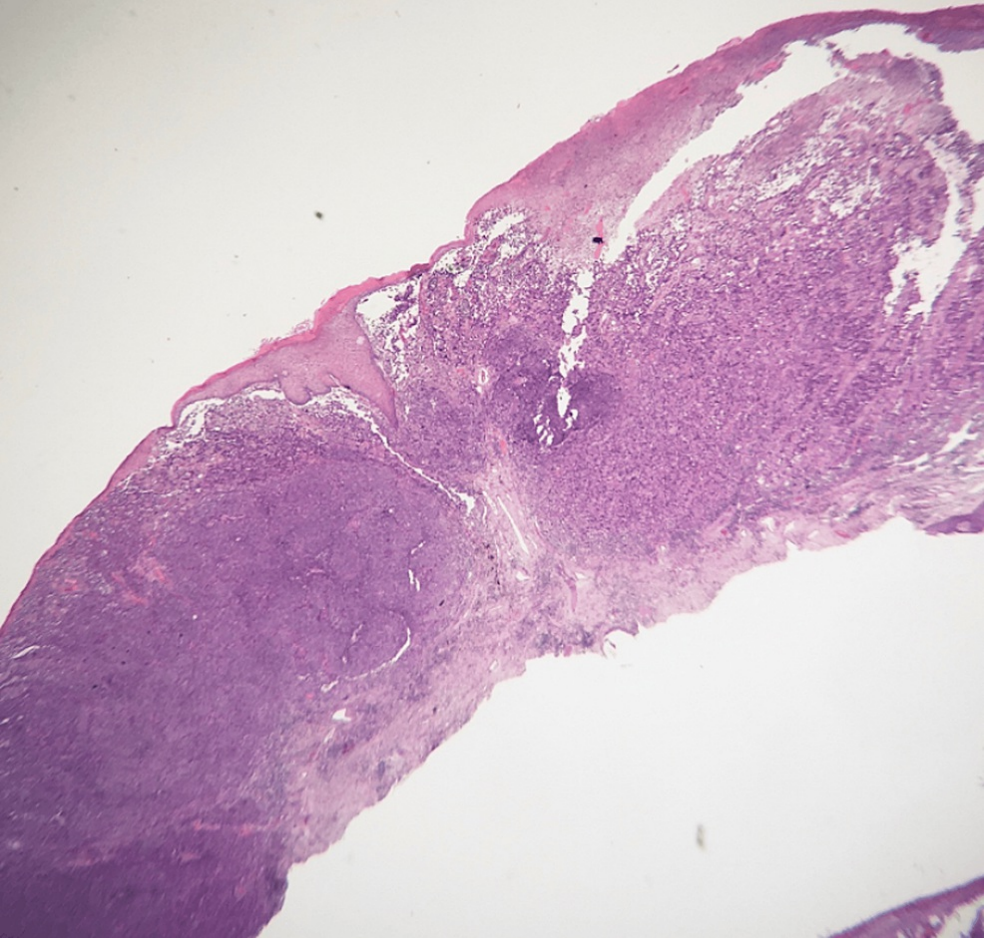
Histologic section of the biopsy showing an ulcerated malignant proliferation with two populations of malignant cells (H&E, magnification 40x).

**Figure 3:**
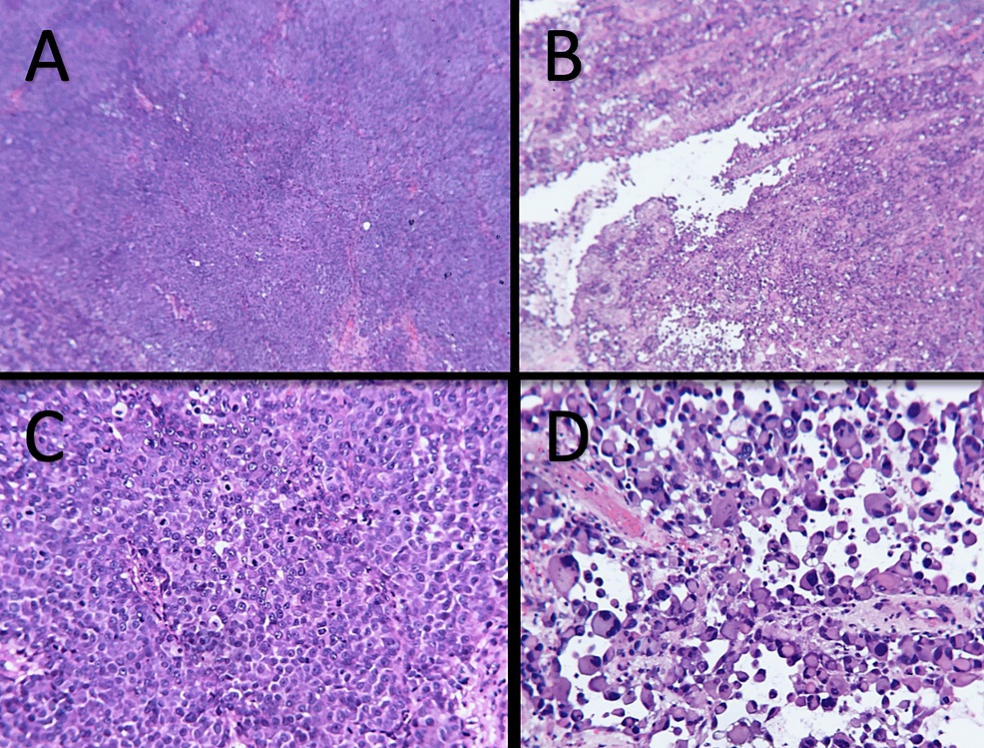
Images A and B demonstrate partially cohesive epithelioid cells with large nucleoli and frequent mitoses (H&E, magnifications 40x). Images C and D show acantholytic large cells with abundant cytoplasm, eccentric nuclei, abundant and atypical mitoses, and papillary projections (H&E, magnification 100x).

On physical exam, a 0.9cm x 0.9cm indurated nodule with central ulceration and a base of confluent erythema was noted on the right posteromedial calf (Figure 1). No other lesions were observed and no lymphadenopathy was detected on exam. An excisional biopsy of the lesion (Figure 2) on hematoxylin and eosin (H&E) stained histologic sections revealed an ulcerated malignant proliferation with two populations of malignant cells. A population of partially cohesive epithelioid cells with large nucleoli and frequent mitoses was observed to merge with an adjacent second population of acantholytic large cells with abundant cytoplasm, eccentric nuclei, abundant and atypical mitoses, and papillary projections (Figure 3). The epithelioid population was positive for Mart-1, HMB45, and SOX-10, supporting the diagnosis of malignant melanoma. Tumor thickness was 3.5mm with ulceration, nodular histology, and absence of perineural invasion. The malignant acantholytic cell population was positive for desmin and calponin, supporting a diagnosis of sarcoma with muscular differentiation. Lymphovascular invasion was identified in the sarcoma component. Both populations were negative for pan-keratin, CD31, and CD34. A diagnosis of a collision tumor involving a malignant melanoma and rhabdomyosarcoma was rendered.

A follow-up PET/CT and MRI brain revealed no evidence of metastatic disease. The patient underwent wide local excision with sentinel lymph node biopsy. No residual melanoma was found in either the excision or lymph node tissue. The patient underwent a repeat PET/CT one month after excision with negative results. At the time of this report, he had started adjuvant pembrolizumab for melanoma stage IIB pT3bN0M0.

## Discussion

We present a unique case of a collision tumor composed of both malignant melanoma and rhabdomyosarcoma. Collision tumors comprise a wide variety of benign and malignant lesions and much regarding their pathogenesis is unknown. However, certain intrinsic and extrinsic factors that contribute to their development have been identified. It is known that severe exposure to sunlight plays a role in the pathogenesis of both melanoma and basal cell carcinoma [3]. In a study of 77 histopathologically confirmed collision lesions, lifetime exposure to UV radiation was also reported as the primary extrinsic factor that correlated with the incidence of collision tumors [2]. Notably, melanocytic components were detected in collision tumors more frequently in individuals under the age of 60, while seborrheic keratosis and basal cell components were more commonly found in individuals over the age of 60 [2]. In addition, collision tumors were more often isolated in males than females (63% versus 43.3%) [2].

Reports describing primary collision tumors involving melanoma and sarcoma are rare. Of the two cases describing the collision of melanoma and sarcoma reported in the literature, both collision lesions were first noted in lymph node metastases [4,5]. It was postulated in these reports that transdifferentiation of malignant melanoma cells to a sarcomatous phenotype could explain the development of a distinct tumor type [4,5]. Melanocytes are embryonically derived from the neural crest. Cells of the neural crest arise from the neuroectoderm and migrate along dorsal and ventral pathways where they differentiate into a variety of tissue types, including smooth muscle, connective tissue, glandular tissues, neurons, glia, and melanocytes [6]. The diversity of cell types to which neural crest cells differentiate and the origin of melanocytes from this population have been posited as factors explaining the relative diversity of oncologic subtypes seen in melanoma. Epithelial-to-mesenchymal-like transition events occur in melanoma that increase the metastatic ability and treatment resistance; melanomas that have undergone this transition often exhibit gene expression profiles that overlap with mesenchymal cancers [7]. Although the exact cause of collision tumors is still unknown, it is believed that chemical signaling produced by an initial tumor may induce modifications in the epithelium or stroma, encouraging differentiation of the second tumor type [1,3]. Accordingly, changes in melanoma differentiation have been noted to occur in response to alterations in the local inflammatory milieu [7]. This case is unique in that the primary collision tumor was not observed in the lymph node, raising the potential of its origin as either a product of melanomatous trans-differentiation or that of two distinct, but locally congruent, oncologic events.

Accurate diagnosis of collision tumors and their components can prove to be challenging due to their rarity and indistinct presentation. Abnormal-appearing skin findings sometimes result from the collision of two benign lesions, leading to unnecessary biopsies [2,8]. However, it is still imperative to examine all quadrants of a lesion for malignant characteristics using both physical exam and dermoscopy [2,8]. Identifying benign characteristics within a lesion and prematurely labeling a lesion as benign could lead to the missed diagnosis of a collision tumor with both benign and malignant components, increasing the risk for metastasis [2,6]. Excision is typically the preferred therapy for the treatment of collision tumors, with the more life-threatening component dictating the treatment and prognosis [3]. Patients should be monitored for five to 10 years after primary treatment to assess for recurrence.

## Conclusions

In this report, we show collision tumors comprise many different cell types, including both benign and malignant cells. This rare case of a collision tumor comprising both malignant melanoma and rhabdomyosarcoma is a reminder that collision tumors take many forms, even if some forms have not yet been described in the medical literature. This rare incidence of malignant melanoma and rhabdomyosarcoma within the same collision tumor requires awareness among dermatologists and dermatopathologists, as patients with these skin lesions may initially present to these specialists for a proper work-up and diagnosis. To properly diagnose a collision tumor, a high index of clinical suspicion must be maintained and a thorough physical examination and history must be taken. Raising awareness about this rare skin lesion will allow proper diagnosis of similar skin lesions in the future.
